# Chemoradiotherapy Strategies for Immunotherapy-Sensitive Multi-Metastatic Nasopharyngeal Carcinoma: A Comparative Case Report and Literature Review

**DOI:** 10.3390/curroncol32080466

**Published:** 2025-08-18

**Authors:** Zikun Li, Yuxiang He

**Affiliations:** Oncology Department, Xiangya Hospital Central South University, Changsha 410008, China; kzllzkkzl@csu.edu.cn

**Keywords:** nasopharyngeal carcinoma, multi-metastasis, immunotherapy-sensitive, palliative radiotherapy

## Abstract

This study investigates two cases of stage IVb de novo multi-metastatic nasopharyngeal carcinoma (NPC) that responded to immunotherapy but resulted in different outcomes. Case 1 involved a multi-metastatic NPC patient (T4N3M1) with extensive bone and lymphatic metastases and severely impaired physical condition (ECOG PS 2) who showed significant tumor reduction after one cycle of immunotherapy combined with non-platinum chemotherapy, with no radiation exposure. Due to financial difficulties, the patient received intermittent immunotherapy plus chemotherapy and survived 28 months with a good quality of life. Case 2 describes a multi-metastatic NPC patient (T3N2M1) with multi-organ (bone and liver) metastases and good performance status (ECOG PS 0) who underwent standard chemotherapy, immunotherapy, and radiotherapy but experienced rapid progression and died after 21 months. Immunotherapy combined with chemotherapy remains the standard for multi-metastatic NPC patients. Patients responsive to induction chemotherapy gain survival benefits from subsequent radiotherapy. However, the advantages and disadvantages of radiotherapy for immunotherapy-sensitive multi-metastatic NPC patients are still unclear. Radiotherapy (RT) can enhance local control and promote tumor antigen release, thereby complementing immunotherapy; yet it can also damage immune cells, leading to exhaustion and resistance. Therefore, balancing RT and chemotherapy is vital for optimizing immune synergy and preventing immune exhaustion.

## Simple Summary

This study investigates the optimal combination of chemotherapy and radiotherapy in de novo multi-metastatic nasopharyngeal carcinoma patients who respond to immunotherapy. It compares two cases with similar responses to immunotherapy but different outcomes: one patient, treated with a less toxic regimen of albumin-bound paclitaxel without radiotherapy, survived 28 months. In contrast, another patient who received standard GP chemotherapy plus radiotherapy died after 21 months, despite initially responding well to immunotherapy. By analyzing these cases and reviewing recent high-quality studies, the study concludes that carefully balancing the intensity of chemotherapy and radiotherapy is crucial for multi-metastatic patients who respond favorably to immunotherapy. While radiotherapy can enhance the immune response, it may also damage immune cells, emphasizing the need to optimize treatment to maximize synergy and prevent immune exhaustion.

## 1. Introduction

Nasopharyngeal carcinoma (NPC) is an aggressive cancer originating in the nasopharynx and is prevalent in East and Southeast Asia. About 10–15% of patients are diagnosed with distant metastasis, while 10–20% may develop it after radiotherapy chemotherapy [1]. First-line GP chemotherapy offers a median overall survival of 22.1 months for recurrent or metastatic NPC. Emerging evidence suggests that immune checkpoint inhibitors extend overall survival (OS) [2,3]. So, combining chemotherapy and immunotherapy is now the standard for recurrent or metastatic NPC. For immunotherapy-sensitive NPC with multiple metastases, preserving immune function while minimizing the potential adverse effects of radiotherapy and chemotherapy is crucial. Immunotherapy is the main approach for these patients to achieve long-term survival. But the monotherapy response rate for second-line treatment in recurrent or metastatic NPC is only about 20% [4]. Chemoradiotherapy (CRT) can significantly increase the response rate of immunotherapy to 80–90% [2,3]. This success, however, brings the role of conventional chemoradiotherapy (CRT) into a more complex and nuanced discussion. This is particularly true for de novo multi-metastatic nasopharyngeal carcinoma, where CRT presents a clinical dilemma due to its dual effects on the immune system. On the one hand, CRT can potentiate the systemic immune response by inducing immunogenic cell death and releasing tumor antigens.

On the other hand, its profound lymphodepleting effects can severely compromise the very effector cells essential for a sustained anti-tumor response. This duality prompts a critical re-evaluation of the traditional treatment paradigm, especially for the specific subpopulation of patients who demonstrate exceptional sensitivity to immunotherapy. It raises the question of a potential paradigm shift: should immunotherapy serve as the foundational “backbone” of therapy, with chemotherapy and radiotherapy carefully calibrated to play a supportive, synergistic role? To achieve this immune synergy, the timing and intensity of these modalities must be precisely individualized. Through the detailed analysis of two representative multi-metastatic NPC cases with starkly different outcomes, this article aims to explore this therapeutic challenge, providing clinical insights into the critical decisions surrounding chemotherapy and radiotherapy for these immunosensitive patients.

## 2. Case 1

**Figure 1:**
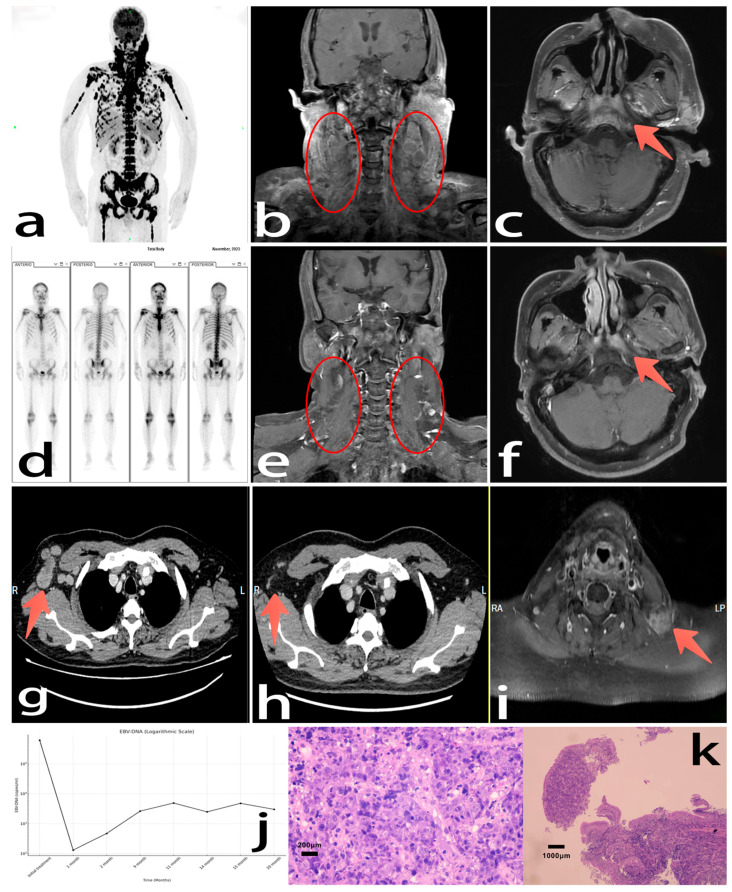
Initial diagnostic imaging: (**a**) Pre-treatment PET-CT showed NPC with extensive skeletal and nodal metastases. (**b**,**c**) Pre-treatment MRI revealed clivus invasion and large-scale fusion of cervical lymph nodes with invasion of muscles and skin. Seven months after two cycles of first-line therapy. (**d**) SPECT showed complete metabolic resolution of skeletal metastases. (**e**) Coronal MRI suggested nodal lesions near-complete recession. (**f**) Axial MRI suggested diminished nasopharyngeal and clivus enhancement. (**g**) A newly detected axillary lymphadenopathy on CT. Fourteen months after diagnosis and five months after the second treatment. (**h**) CT showed complete regression of the right axillary lymphadenopathy. (**i**) MRI showed a new nodal lesion in the left cervical region. (**j**) EBV-DNA load fluctuations: EBV-DNA copies dropped rapidly after two cycles of treatment, slightly increased, and remained relatively low until the last follow-up date. (**k**) Pathological results suggested an undifferentiated non-keratinizing carcinoma.

A 51-year-old male was diagnosed with NPC, with bilateral cervical lymphadenopathy as the initial symptom. After two months, he experienced breathing difficulties and general weakness, losing over ten pounds in weight with no previous history of other malignancies or infectious diseases; however, his mother had a history of gastric cancer. Examination revealed huge, fixed, hard cervical lymph nodes with skin adhesion, as well as a 2 × 3 cm right axillary node, and severe dyspnea, with a PS score of 2. Pathological results indicated undifferentiated non-keratinizing carcinoma, EBER (++), and a PD-L1 combined positive score (CPS 40); PET-CT and MRI imaging showed extensive lymph node involvement, skeletal metastases, and possible lung metastases (Figure 1a–c). The cancer was staged as T4N3M1 IVb (AJCC 8th). Laboratory tests revealed anemia (Hb 61 g/L), thrombocytopenia (43 × 10^9^/L), elevated lactate dehydrogenase (LDH) (6029 U/L), and Epstein–Barr virus (EBV) DNA (6.15 × 10^5^ copies/mL). Liver and kidney functions were generally normal. After informed consent, the patient immediately received nab-paclitaxel (400 mg Day 1) + tislelizumab (200 mg Day 1) + capecitabine (1.5 g bid Days 1–14), achieving rapid symptomatic relief within a week. One month later, EBV-DNA reduced to 1.31 × 10^2^ copies/mL and LDH 332 U/L. He felt very well and continued the same protocol, even returning to work after the second cycle.

Then, due to financial difficulties, this patient was hospitalized seven months later. The examination for evaluating the therapeutic effect showed the primary lesion had been well controlled: SPECT demonstrated no metabolic activity of the primary skeletal metastases (Figure 1d); MRI showed significant regression of primary and cervical lesions approaching complete clinical remission (Figure 1e,f). But thoracoabdominal CT revealed a new 24 mm axillary node (Figure 1g), with EBV-DNA levels rising to 2.63 × 10^3^ copies/mL. Then, the patient received two cycles of the combined regimen: nab-paclitaxel (400 mg, Day 1) + cisplatin (75 mg, Days 1–2) + tislelizumab (200 mg, Day 1). Then, he stopped treatment for as long as five months due to financial difficulties. Upon returning to the hospital, a CT scan showed that the axillary nodes had resolved (Figure 1h). Nasopharyngeal MRI and physical examination indicated left cervical nodal progression (Figure 1i). After two cycles of the original treatment regimen, physical examination revealed that the left cervical node had disappeared. The patient was subsequently followed up every three months without any drugs for 7 months without any medications for 7 months. The final follow-up was 28 months after the initial diagnosis. The disease remained controlled, and the patient could perform routine work and daily activities remarkably. Figure 1j shows the changes in the EBV-DNA copy number of the patient during treatment and follow-up. Figure 1k is pathological specimen image of the patient under the microscope.

## 3. Case 2

**Figure 2:**
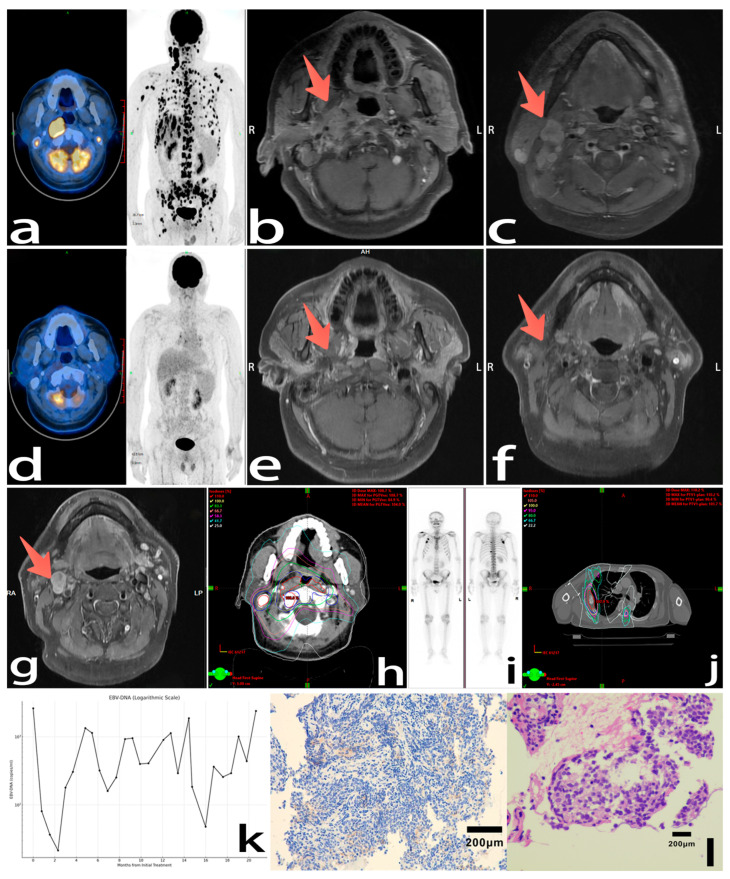
Initial diagnostic imaging: (**a**) Pre-treatment PET-CT demonstrated NPC with widespread metastatic involvement of bone and liver. (**b**,**c**) Pre-treatment MRI delineating nasopharyngeal primary and cervical nodal metastases. Post-first-line treatment evaluation: (**d**) post-six-cycle systemic therapy PET-CT showed metabolic resolution of primary and metastatic tumors (SUVmax 2.5 vs. baseline 22.6). (**e**,**f**) MRI-confirmed complete response (CR) with nasopharyngeal lesion and cervical lymph nodes. Post-second-line treatment evaluation: (**g**) Progressive right cervical nodal enlargement with necrotic rim sign. (**h**) Radiation dosimetry map for primary site palliative RT. (**i**) SPECT-confirmed osseous progression. (**j**) Metastatic rib lesion radiation planning. (**k**) EBV-DNA load fluctuations: EBV-DNA copies dropped remarkably but soon rose and remained relatively high until the last follow-up. (**l**) Pathological results suggested an undifferentiated non-keratinizing carcinoma.

A 59-year-old male presented with right cervical lymphadenopathy and headache, without weight loss or decreased appetite. The patient denied any chronic comorbidities, infectious diseases, or previous malignancies. The PS score was 0. A nasopharyngeal biopsy confirmed the presence of non-keratinizing squamous carcinoma, characterized by an immunohistochemical profile of EBER (+), PD-L1 (CPS 5, TPS 2%). The patient’s serum EBV-DNA level was 2.62 × 10^3^ copies/mL, and LDH was normal before treatment. PET-CT imaging revealed a hypermetabolic nasopharyngeal mass with associated right cervical nodal, hepatic, and multiple skeletal metastases (Figure 2a). MRI of the nasopharynx and neck indicated nasopharyngeal tumor invasion into the right tensor/palatal muscles and clivus (Figure 2b,c). The cancer was staged as T3N2M1 IVb (AJCC 8th).

He received the standard treatment recommended by the guidelines: six cycles of the GP regimen, gemcitabine (1.8–1.9 g Days 1, 8) and cisplatin (70–90 mg Days 1–2), in combination with tislelizumab (200 mg Day 1) every three weeks for first-line treatment. This regimen achieved cCR after six cycles: PET-CT suggested that the primary tumor and hepatic or bone lesions had disappeared (Figure 2d). MRI scans also confirmed near-complete regression of nasopharyngeal and nodal lesions (Figure 2e,f). Subsequently, maintenance therapy was initiated with tislelizumab (200 mg) and capecitabine (1.5 g bid Days 1–14, every 3 weeks). After four months of maintenance therapy, a head and neck MRI revealed enlargement of a right level II cervical lymph node (short axis 12 mm, previously 6 mm) and an increase in EBV-DNA copies. Nab-paclitaxel (200 mg Day 1) was administered for four cycles. A follow-up MRI showed that the lymph node remained stable at 12 mm. After another six months of tislelizumab and capecitabine maintenance therapy, the MRI indicated thickening of the nasopharynx left lateral wall and slight enlargement of the previously noted right level II cervical lymph node (14 mm), with enhancement and central necrosis (Figure 2g). The CT scan of the chest, abdomen, and pelvis indicated no other distant metastasis had progressed. Meanwhile, the patient’s general condition was acceptable, and no complications such as grade 3-4 bone marrow suppression had occurred. So, the nasopharynx local and regional palliative radiotherapy were planned; only residual primary and nodal tumors and the metastatic lesions in the cervical vertebrae (PGTVnx/nd 60 Gy/30 fractions, PGTVm 45 Gy/25 fractions; Figure 2h) were included in the irradiation field. Weekly cisplatin (75 mg) concurrent chemotherapy was carried out for three cycles, and then it was maintained with tislelizumab and capecitabine.

Three months after RT, the patient complained of chest pain and experienced a loss of appetite and fatigue. SPECT identified new metastatic lesions in the sternum and ribs (Figure 2i). Subsequently, the rib lesions were treated with radiotherapy to relieve pain (PGTVm 22.5 Gy/5 fraction, Figure 2j). At the same time, maintenance therapy was switched to toripalimab (240 mg Day 1) and capecitabine in three-week cycles. A nasopharyngeal MRI showed significant improvement in the primary lesions and lymph nodes three months after RT. However, EBV-DNA and LDH levels rose further. The regimen was changed to camrelizumab (200mg Day 1) and anlotinib (8 mg Days 1–14) for two months. During this period, EBV-DNA copies increased, reaching a peak of 2.40 × 103 copies/mL, suggesting ongoing progression. Furthermore, after RT, the patient suffered from progressively worsening myelosuppression (white blood cells, platelets, and red blood cells all decreased by 2–3 degrees). Figure 2k shows the changes in the EBV-DNA copy number of the patient during treatment and follow-up. Figure 2l is a pathological specimen image of the patient under the microscope. Ultimately, the patient succumbed to infection and multi-organ failure 21 months after the initial diagnosis.

## 4. Discussion

The two nasopharyngeal carcinoma cases both involved extensively metastasized lesions, and both were highly sensitive to first-line immunotherapy and chemotherapy. However, they ultimately yielded different results (Table 1). The primary reason for these differing treatment outcomes lies in the intrinsic biological behavior of the tumors. Nevertheless, variations in treatment modalities—such as differences in chemotherapy intensity and the administration of radiotherapy—may also significantly influence the efficacy of immunotherapy. These two cases compel us to reflect on the following issues:

Firstly, in the era of immunotherapy, the prognosis for patients with metastatic cancer cannot be determined solely by traditional indicators such as clinical stage, tumor burden, or general condition. The most crucial factor is how the tumor responds to immunotherapy. For patients who are sensitive to immunotherapy, regardless of disease stage, the tumor can be controlled rapidly and durably, just like in Case 1. The patient in Case 1 had a significantly higher CPS score than the patient in Case 2, suggesting that immunotherapy may not have played as pivotal a role in the favorable outcome of Case 2 as it did in Case 1.

Secondly, how can we identify patients who may benefit from immune checkpoint inhibitors? Unfortunately, there are no ideal markers for prognosis prediction. PD-L1 expression is the most used biomarker in practice; however, the predictive value of PD-L1 expression for PD-1 blockade remains controversial for NPC. In KeyNote 122, it is suggested that patients with CPS less than 10 are likely to benefit from PD-1 blockade. Meanwhile, in Jupiter-02 and Rational 309, patients with high and low PD-L1 expression could benefit from PD-1 blockade. However, the CONTINUUM trial indicates that patients with high CPS are more likely to benefit from PD-1 blockade. In the real world, most patients with high PD-L1 expression achieved more profound and lasting remission. Case 1 exhibited a high level of PD-L1 expression (CPS 40) and was exceptionally responsive to immune checkpoint blockade. In Case 2, the patient also showed a favorable response to first-line chemo-immunotherapy; however, the duration of remission was relatively short. This may be attributed to the low PD-L1 expression level (CPS = 5, TPS 2%). As a result, the anti-tumor effect was not sustained.

Thirdly, is the GP regimen the best chemotherapy when used together with immune checkpoint inhibitors (ICIs) for the immune-advantaged population? A study by Yi-Feng Yu suggests that the induction chemo-immunotherapy of camrelizumab plus modified TPF demonstrates an excellent CR rate and an acceptable safety profile in patients with locoregionally advanced nasopharyngeal carcinoma (LANPC) [5]. Low-dose, low-toxicity chemotherapy combined with PD-1 blockade may enhance immunotherapy effects while preserving immune capacity, achieving synergy and minimizing toxicity [6,7,8]. This relates to modulating the immune microenvironment. Low-dose chemotherapy (e.g., albumin-bound paclitaxel) decreases immune-suppressive cell infiltration (such as regulatory T cells), promoting tumor antigen release and dendritic cell maturation, thereby improving the efficacy of PD-1 monoclonal antibodies.

In treating metastatic nasopharyngeal carcinoma with multiple distant metastases, what is the role of radiotherapy, and does it apply to patients sensitive to immunotherapy?

The progression pattern observed in Case 1 suggests that if progression consistently shifts to new sites after systemic therapy, irradiating the primary tumor may be futile.

In You Rui’s research, local-regional radiotherapy of the nasopharynx can further enhance survival for patients with a partial or complete response to three cycles of PF chemotherapy. The 24-month OS was 76.4% in the chemotherapy plus radiotherapy group, compared with 54.5% in the chemotherapy alone group [9]. The rationale for RT at the primary site originates from the PF regimen era, but no definitive evidence supports its benefit with GP chemotherapy or GP-based chemo-immunotherapy. The study also did not stratify patients by oligometastatic versus widespread metastatic status. Patients with multiple metastatic lesions have shorter survival times than those with one lesion or fewer than three lesions. A small-sample (*n* = 51), prospective, single-arm, single-center study indicated that patients with newly diagnosed oligometastatic NPC can attain long-term survival after undergoing radical radiotherapy to the primary tumor and local treatment for metastases after platinum-based palliative chemotherapy. The 1-year, 3-year, and 5-year PFS and OS rates were 76.5%, 38.1%, and 31.8% and 98%, 75.4%, and 45.6%, respectively [10]. Radiotherapy targeting the primary tumor or both primary and metastatic lesions can improve survival in chemotherapy-sensitive or oligometastatic nasopharyngeal carcinoma. However, neither study involved immunotherapy.

Researchers recently introduced the “6th R of Radiobiology”—reactivating anti-tumor immune responses [11]. This concept may reconcile the interplay between RT and immunotherapy. RT can cause immunogenic cell death, enhancing tumor cell visibility to the immune system and activating specific anti-tumor responses. However, RT also promotes immunosuppressive mechanisms that can limit anti-tumor immunity by altering the TME (tumor microenvironment). It can increase programmed death-ligand 1 (PD-L1) expression on tumor and immunosuppressive myeloid cells and elevate T-cell immunoreceptors with Ig and ITIM (T-cell immunoglobulin and immunoreceptor tyrosine-based inhibitory motif) domains. The effectiveness of RT-induced anti-tumor responses relies on the balance between immune activation and immunosuppression, influenced by RT fractionation schedules. RT alone usually cannot generate a lasting systemic immune response. Studies indicate that combining RT with immunotherapy, especially ICIs, shows promise. A recent study highlighted that radiotherapy plays a significant role in immune modulation in NPC. This mechanism is associated with the activation of dendritic cells, which in turn enhances the effects of tumor immunotherapy [12]. In managing de novo metastatic nasopharyngeal carcinoma (dmNPC), numerous research teams have investigated integrating RT with immunotherapy.

A multicenter propensity score-matching study assessed the effectiveness of local-regional radiotherapy (LRRT) in patients with dmNPC undergoing chemo-immunotherapy. While the multivariable analysis revealed that LRRT was not an independent prognostic factor in the matched cohort, subgroup analysis indicated a notable improvement in progression-free survival (PFS) for patients with oligo metastatic disease (OMD) who underwent LRRT (3-year PFS rate: 70.6% vs. 49.3%, *p* = 0.043). Conversely, no similar advantage was observed in patients with poly-metastatic disease (PMD), whose 3-year PFS rates were 35.8% compared to 27.8%, *p* = 0.17 [13]. But the therapeutic benefits of radiation therapy are particularly pronounced in patients exhibiting oligometastatic disease, Epstein–Barr Virus (EBV) DNA levels below 20,200 copies/mL, and those who have experienced either complete or partial relapse following immunochemotherapy [14].

In both reported cases, the extensive, multifocal metastatic disease and exceptionally high tumor burden made radiotherapy to either the primary lesion or the metastases unlikely to confer a survival benefit. Therefore, maintenance treatment with immunotherapy plus capecitabine should be initiated once remission is achieved with induction therapy.

To investigate the abscopal efficacy of metastasis-directed therapy (MDT), MDT (27 Gy/3 fr) was added to camrelizumab (Cam) in patients with recurrent or metastatic nasopharyngeal carcinoma (R/M-NPC). The results did not meet the primary endpoint of superior ORR of unirradiated lesions with the addition of MDT to Cam [15]. Thus, for metastatic NPC, survival benefits from radiotherapy are likely confined to oligometastatic disease treated with focal irradiation, whereas no significant improvement is observed in poly-metastatic settings.

The area of the irradiated field can affect the immunomodulatory properties of RT. A small-sample (*n* = 45) study indicated that irradiation was only delivered to the primary tumor, retropharyngeal nodes (GTVnx+rn), and gross cervical lymph nodes (GTVnd). Omitting CTV1 and CTV2 was well tolerated and provided favorable clinical outcomes in the era of immunotherapy [16].

In case 2, oligo-progression lesions were observed in the cervical lymph nodes, nasopharyngeal, and rib areas. Palliative radiotherapy was first administered targeting the progressive primary lesions and the metastatic cervical spine lesions based on MRI (PGTVnx/nd 60 Gy/30 fr). Then, the rib lesions were treated with radiotherapy to relieve pain (PGTVm 22.5 Gy/5 fr) and trigger an abscopal efficacy. Unfortunately, a deterioration in performance status was observed, and myelosuppression worsened following palliative radiotherapy. This might be related to the excessive irradiation of the nasopharyngeal and cervical lymphatic drainage areas. Although no CTVs were planned, the volume of GTVnx/nd based on MRI, not PET-CT, may have been too large (as large as 200cc).

Further research is needed to explore treatment modalities for patients with immunotherapy advantages and the optimal radiation field and dose for these individuals.

## 5. Conclusions

While the integration of immunotherapy with chemotherapy is the established first-line standard for multi-metastatic NPC, detailed subgroup analyses remain scarce. Specifically, there is little high-level evidence to guide the optimal management of patients with a high metastatic burden who also demonstrate exceptional sensitivity to immunotherapy. The strategy regarding subsequent radiotherapy to the primary site and cervical lymph nodes remains a topic of clinical discussion, especially for the subgroup of patients who exhibit sensitivity to immunotherapy. Our report, by presenting two such cases with divergent outcomes, aims to contribute to this discussion. We argue that for the unique subpopulation of patients with de novo, multi-metastatic disease and a clear immunotherapy advantage, a fundamental re-evaluation of the treatment hierarchy is warranted. In this context, systemic therapy should remain predominant, with immunotherapy serving as the primary therapeutic modality. Radiotherapy and chemotherapy would act as synergistic partners to enhance immunogenicity. This approach necessitates a conscious effort to minimize the radiation field and dose, particularly in lymphatic regions, to preserve crucial lymphoid effector cells and the patient’s overall immune competence. The goal of radiation should not be to achieve radical treatment but rather to complement the systemic immune response. Ultimately, our findings underscore that for patients with extensive metastatic disease who are highly responsive to immunotherapy, the timing, method, and even necessity of radiotherapy must be carefully individualized. Further investigation is essential to help refine treatment strategies for this important, albeit specific, patient cohort.

## Figures and Tables

**Table 1:** This table presents the clinical timelines of two patients with extensively metastatic NPC. Note: ECOG PS, Eastern Cooperative Oncology Group Performance Status; LDH, lactate dehydrogenase; EBV-DNA, Epstein–Barr virus DNA; PD-L1, programmed death-ligand 1; CPS, combined positive score; GP, gemcitabine plus cisplatin; cCR, complete clinical remission; PET-CT, positron emission tomography/computed tomography; RT, radiotherapy.

Comparative Timeline of Two Cases		
Phase of Treatment	Patient 1 (Survived 28 Months)	Patient 2 (Died at 21 Months)
Diagnosis and baseline	Age: 51, male. Stage: T4N3M1 (extensive nodal, bone, lung metastases). Condition: poor (ECOG PS 2), severe dyspnea, anemia, thrombocytopenia. Biomarkers: high tumor load (LDH 6029, EBV-DNA 6.15 × 10^5^), PD-L1 (CPS 40).	Age: 59, Male. Stage: T3N2M1 (hepatic, bone metastases). Condition: good (ECOG PS 0). Biomarkers: normal LDH, EBV-DNA 2.62 × 10^3^, PD-L1 (CPS 5).
Initial treatment and response	Regimen: nab-paclitaxel + tislelizumab + capecitabine. Response: rapid symptomatic relief; EBV-DNA and LDH dropped dramatically. Returned to work after second cycle.	Regimen: standard GP (gemcitabine + cisplatin) + tislelizumab (6 cycles). Response: achieved complete clinical remission (cCR) confirmed by PET-CT.
First progression and management	Event: after 7 months of inconsistent treatment due to financial difficulties, a new axillary node progressed. Management: received two cycles of a stronger regimen (added cisplatin).	Event: after 4 months of maintenance therapy (tislelizumab + capecitabine), cervical node progressed. Management: received four cycles of nab-paclitaxel; node remained stable.
Second progression and management	Event: after another 5-month treatment gap, progression in a different area (cervical node). Management: re-treated with the original regimen; node disappeared.	Event: after 6 more months of maintenance, further progression in nasopharynx and cervical node. Management: palliative radiotherapy (RT) to nasopharynx and nodes with concurrent weekly cisplatin.
Late-stage events and key divergence	Follow-up: subsequently followed for 7 months without any medication. Radiotherapy: never received radiotherapy. Immune status: remained enough to control disease with intermittent therapy.	Post-RT: 3 months after RT, developed new, painful bone metastases. Radiotherapy: received another course of palliative RT for pain. Immune status: suffered from progressively worsening myelosuppression post-RT.
Outcome	Survived 28 months with good quality of life and ability to work.	Succumbed to infection and multi-organ failure at 21 months.

## Data Availability

The raw data supporting this article’s conclusions are derived from the patients’ medical records. Due to the sensitive nature of this information and to protect patient privacy, the dataset is not publicly available. Requests for de-identified data can be directed to the corresponding author upon reasonable request and subject to institutional and ethical approvals.
